# Active Angular Alignment of Gauge Blocks in Double-Ended Interferometers

**DOI:** 10.3390/s131013090

**Published:** 2013-09-27

**Authors:** Zdeněk Buchta, Šimon Řeřucha, Václav Hucl, Martin Čížek, Martin Šarbort, Josef Lazar, Ondřej Číp

**Affiliations:** Institute of Scientific Instruments, Academy of Sciences of the Czech Republic, Královopolská 147, Brno 612 64, Czech Republic; E-Mails: res@isibrno.cz (Š. Ř.); treak@isibrno.cz (V.H.); cizek@isibrno.cz (M.Č.); sarbort@isibrno.cz (M.Š.); joe@isibrno.cz (J.L.); ocip@isibrno.cz (O.Č.)

**Keywords:** low-coherence interferometry, gauge block, metrology

## Abstract

This paper presents a method implemented in a system for automatic contactless calibration of gauge blocks designed at ISI ASCR. The system combines low-coherence interferometry and laser interferometry, where the first identifies the gauge block sides position and the second one measures the gauge block length itself. A crucial part of the system is the algorithm for gauge block alignment to the measuring beam which is able to compensate the gauge block lateral and longitudinal tilt up to 0.141 mrad. The algorithm is also important for the gauge block position monitoring during its length measurement.

## 1. Introduction

In the field of industrial metrology, a gauge block stands for a length standard [1]. The gauge block is used there for verifying of the length measuring instruments used in all branches of mechanical manufacturing. Like all other mechanical measuring tools, gauge blocks need to be calibrated periodically. At present, their calibration methodology is described in the international regulation EN ISO 3650. The methods described there are based on using of mechanical length gauges or laser interferometry [2,3], but none of them is a full contactless method.

From a practical point of view, techniques used nowadays might influence the measured length value due to a mechanical contact between the gauge block and a part of the measurement setup. Therefore, the research in the field of contactless calibration techniques is been pursued incessantly.

Most contactless measuring techniques are based on a double-ended interferometry principle where different kind of lights (white, monochromatic, coherent) are used for detection of the gauge block length [4–8]. Most of these techniques are based on some changes of the optical setup (*i.e.*, using shutters for disabling some beams) during the measuring process. Previously, we put together a contactless method published in [9] combining laser interferometry and low-coherence (white-light) interferometry [10,11]. In this case, the contactless measurement of the absolute gauge block length is done as a single-step operation without any change in optical setup during measurement, giving complete information of the gauge block length. The contactless method employing light for the object length measurement eliminates the measurement error caused by a mechanical interaction between the object and the measurement setup. On the other hand, the precise measurement is conditioned by perfect alignment of the block-shaped object (gauge block) to the measuring beam. If the axis of the gauge block is not parallel with the beam axis, the result of the measurement is influenced by a cosine error. Elimination of the cosine error requires employing of a powerful control technique ensuring the proper object positioning in the experimental setup during its length measurement. This article describes the technique which was designed for our system for contactless gauge block length measurement.

## 2. Method for Contactless Gauge Block Length Measurement

**Figure 1.**
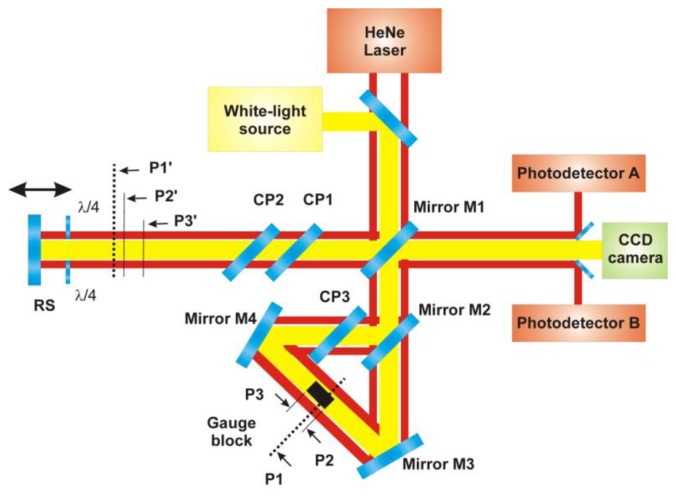
Optical setup for gauge blocks measurement. CP1, CP2 and CP3 are compensating plates and RS is a reference surface.

The optical setup combines a Michelson interferometer and a Dowell interferometer [12], placed in the reference arm of the Michelson interferometer. The principle of the measurement is illustrated in Figure 1 and described in detail in [9,13]. A parallel beam, generated by a broad-band (e.g., white-light) source, is divided into two parts by semireflecting mirror M1. The resulting measuring beam goes through a couple of compensating plates CP1 and CP2 and after that is reflected back by a reference surface RS.

The resulting reference beam of the Michelson interferometer plays the role of the primary beam for the Dowell interferometer. This beam is divided by mirror M2 into two beams going in the Dowell interferometer in opposite directions. One of them passes through compensating plate CP3 and is reflected by mirror M4 onto a gauge block face. The other beam passes through the beamsplitter (mirror M2) and then is reflected by mirror M3 onto the other face of the gauge block. One part of both mentioned beams illuminating the gauge block is reflected back by the gauge block faces and the other part of both beams pass alongside the gauge blocks.

At the output of the Michelson interferometer, there are five beams which could possibly interfere—the first one is the measuring beam of the Michelson interferometer reflected by the reference surface RS, then there is a pair of beams reflected by the gauge blocks and a pair of beams that pass alongside the gauge blocks.

The principle of the measurement is based on low-coherence interferometry taking advantage of low-coherence properties of the broadband light source. In the range of the movable reference surface RS shift, there are three positions where the white-light beams interfere. In the block diagram of the experimental setup shown in Figure 1, these positions are marked as P1′, P2′ and P3′.

In the P1′ position, the Michelson interferometer measuring beam interferes with the pair of beams passing alongside the measured gauge block. In fact, this is equivalent to a configuration with a mirror in a position marked as P1 (see Figure 1). P1 is at the mean optical path length of the ring interferometer and for the gauge block length measurement, it plays the role of the reference position.

As for positions P2′ and P3′, the Michelson interferometer measuring beam interferes with the beams reflected by the gauge block faces (marked as P2 and P3 in Figure 1). Then, the measured gauge block length is equal to the sum of distances between the measuring positions P2′ and P3′ of the reference position P1′ [Equation (1)]:

(1) GBL=|P1′−P2′|+|P1′−P3′|

For incremental interferometric measurement of the distance between the reference position P1 and measuring positions P2 and P3, the red HeNe laser radiation is used. In the previous version (see Figure 1) [12], the incremental interferometric measurement is realized by a couple of laser interferometers (designated A and B) working separately from the white-light interferometer.

## 3. Principle of Gauge Block Position Detection

The measuring axis of the gauge block in the interferometer should be kept in a strictly parallel position with the propagating beams in the Dowell interferometer. If the condition is not ensured then Abbe errors will occur and a reliable measurement of the gauge block length is impossible.

**Figure 2.**
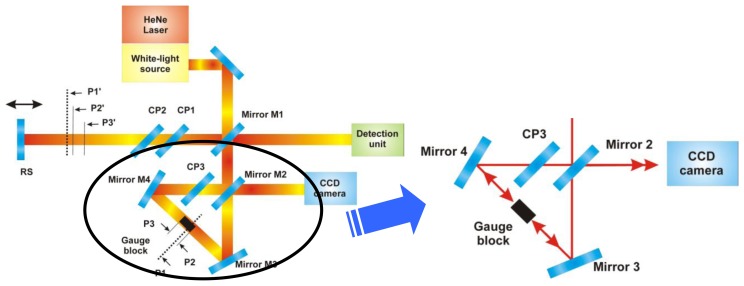
New version of the system for gauge block calibration (left hand side) and a simplified detail view of the dowel interferometer with a gauge block (without the part of the beam going alongside the gauge blosk) and CCD camera used for the gauge block angular alignment. CP1, CP2 and CP3 are compensating plates and RS is a reference surface.

The solution of the problem can be employed by the interference of reflected beams coming from both sides of the inspected gauge block (see Figure 2). We assume a coherent light instead of the white light in this case. If the gauge block is in the right parallel position to the propagating coherent beams in the Dowell interferometer then we observe a surface interference fringe at the unused output of the Mirror M2. If the block is slightly misaligned from the parallel position then the numbers of surface fringes increase or the interference pattern is not visible.

In the previous optical setup of the contact-less interferometer shown in Figure 1, the low-coherent beams illuminate the both sides of the gauge block. Thanks to the larger length difference between these two arms of the Dowel interferometer the low-coherent interference isn't created at the output of the mirror M2. Unfortunately, the incremental interferometers A and B shown in Figure 1 go alongside the gauge block in the Dowell part of the double-ended interferometer. This configuration doesn't allow the possibility of detecting the correct position of the inspected gauge block in the interferometer by an interferometric technique.

In the new approach of the complex optical setup shown in Figure 2, a single beam combining red HeNe radiation and white-light source radiation is used [14,15]. Using of a large single beam integrates coherent laser interferometer and white-light interferometer into a single measuring system working under the exactly same conditions. The beam of the single-mode laser is combined by an optical fiber coupler with the broadband light beam. In the presented system, a broad-band near infrared radiation generated by supercontinuum NKT laser (1.5 W SuperK Extreme Versa, NKT Photonics, Denmark) is used.

An inspection CCD camera records the interference fringes structure given by the two reflected beams from the gauge block sides. On basis of the interference pattern an algorithm for active stabilization of the gauge block can be solved (described in Section 4, shown in Figure 5).

**Figure 3.**
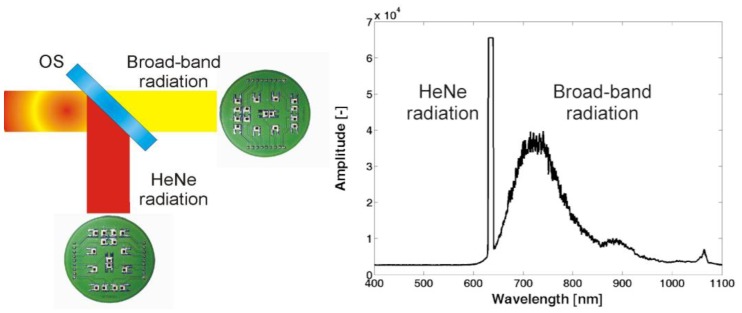
Detail view of the detection unit used in the designed measuring system. *OS* is an optical splitter. On the right hand side, there is shown a spectrum profile of the combined laser beam taken at the input of the detection unit.

The detection chain of the new complex system for the contact-less measurement of the gauge block length has to be modified to be able to split the single mode and broad-band radiation. In the new version, the 633 nm laser radiation and the broad-band radiation are separated by a specially designed and made optical splitter. In combination with a couple of photodetector arrays, the splitter form a detection unit used in the presented system (Figure 3).

In combination with a photodetector array, the beam combining both types of radiation can be spatially divided into a number of autonomous interferometers giving the information about the relevant part of the measuring object and the measuring system itself over the whole beam diameter.

The array of photodetectors used in the HeNe radiation way detects interference fringes generated by the movement of the reference mirror RS of the contact-less interferometer. On basis of the digital signal processing we obtain similar values as from two incremental interferometers A and B in the previous presented setup (Figure 1).

## 4. Algorithm of the Active Stabilization of Gauge Block in the Interferometer

**Figure 4.**
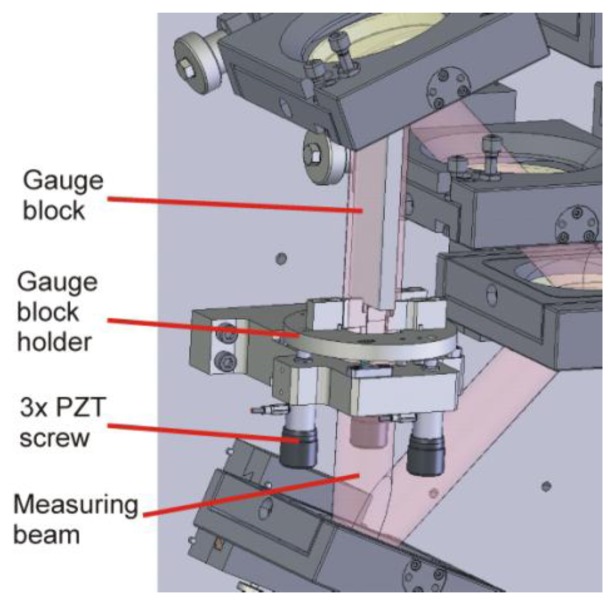
Sketch of the gauge block holder placed in the optical setup.

In a double-ended optical measurement of a gauge block length, the proper position of the gauge block in the measuring system is a critical parameter [1,16]. Before the length measurement, the gauge block is placed by an automatic handling system into a gauge block holder, a tripod driven by PZT screw actuators (PE4, Thorlabs, Newton, NJ, USA) used for gauge block adjustment in the optical setup (see Figure 4). Because of the length measurement principle based on direct optical double-side measurement, the gauge block has to be set to be parallel with the measuring beam. Assuming intact gauge block, its perfect alignment to the measuring beam axis gives in result the right information about the gauge block length. Otherwise, the gauge block length measurement is influenced by the cosine error.

**Figure 5.**
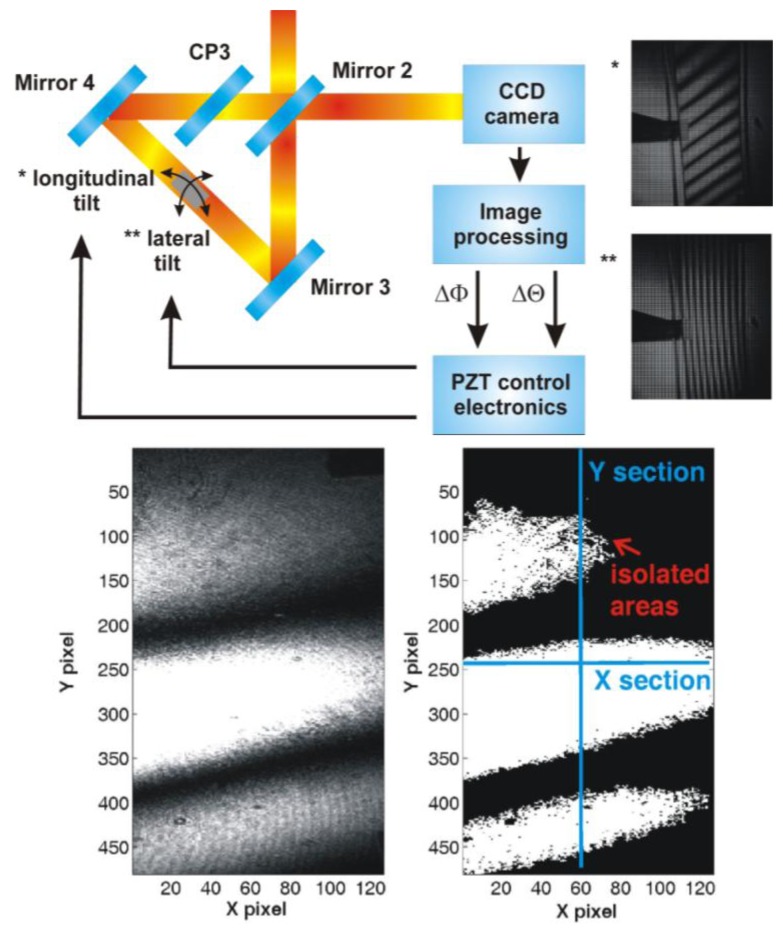
An example of the longitudinal (*) and lateral (**) tilt of the gauge block is shown on the right-hand side of the picture. At the bottom part, there is an example of the recorded image before (left) and after (right) applying of the flooding technique. The picture shows below an example of taking the X and Y sections to identify the number of interference fringes and their orientation.

For the gauge block position analysis, the designed algorithm takes advantage of the large beam used for the gauge block length measurement. At the output of the triangle (Dowell interferometer) in the reference arm of the interferometer, a commercially available USB CCD camera—DFK 41BU02 produced by The Imaging Source Europe GmbH (Bremen, Germany)—is installed (see Figure 2). When the gauge block is placed into the holder, the control software takes a picture from the camera and do the analysis of the image section which shows interference of the HeNe radiation presented in two beams—the one reflected on a bottom surface of the gauge block and the other one reflected on the top of the gauge block paced into the triangle (see Figure 5).

The image analysis employs the “flooding technique” working with gray scale images [17]. In the first step, the value of the mean intensity in the investigated area is determined. Then, the value is used as a threshold separating areas of higher and lower intensity. The resulting image is then analyzed again to identify of areas representing interference fringes—minor areas (up to 100 pixel clusters) are regarded to be a noise and they are not taken into account. Finally, the image analysis procedure gives a number of identified interference fringes in the longitudinal and lateral direction (Figure 5). If the number is greater than 1 or if the number of interference fringes cannot be evaluated, the software readjusts the voltage on an adequate PZT transducer built into the gauge block holder to optimize the gauge block position in the system. For each image, several X and Y sections are analyzed for clear identification of all interference fringes and their orientation. For cases of gauge blocks with shape imperfections, the number of iterations is limited to 10. Then, the software carries out the length measurement or marks the gauge block as defective.

The hardware components of the servo-loop chain realizing the gauge block holder positioning are AD/DA converter and high-voltage amplifier. The AD/DA card is equipped with three analog outputs, each connected to 18-bit DA converter. Then, the signal is amplified by the high-voltage amplifier to cover the range from 0 V to 150 V for driving of the PZT transducer. The used electronics were designed at our institute. Communication between a personal computer and the electronics is ensured by a CAN bus.

In the output of the triangular optical setup, the relation between the mutual angle of interfering beams axis and a period of a spatial interference structure is in case of two-beam interference given by Equation (2):

(2) $$P=\frac{\lambda }{2\cdot \sin(\alpha )}=\frac{\lambda }{2\cdot \sin(4\cdot \beta )}$$

where *P* is a period of a spatial interference structure (period of interference fringes), λ is a laser radiation wavelength and α is the half of mutual angle of interfering beams axes and β is the angular change of the gauge block placed in the triangular optical setup (Figure 5). In the designed experimental setup, the gauge block holder contains PZT transducers with a maximal stroke of 15 μm, which allows alignment of the gauge block axis to the measuring beam axis in a range of tenth of milliradians.

For the pilot experiment, a reference gauge block of 23 mm length was used. The maximal number of interference fringes generated by the PZT induced longitudinal tilt was 46 (gauge block length 30 mm, adequate period of interference fringes *P_longitudinal_* = 0.652 mm), which is adequate for the angular shift of 0.121 mrad (0.007°). The maximal number of interference fringes generated by the PZT induced lateral tilt was 16 (gauge block width 9 mm, adequate period of interference fringes *P_lateral_* = 0.563 mm), which is adequate to the angular shift of 0.141 mrad (0.008°). This range is regarding to be sufficient for compensation of thermo-mechanical drifts of the gauge block holder (stainless steel, thermal expansion coefficient 10.6 × 10^−6^ K^−1^) used in standard laboratory conditions.

As described above, the algorithm for the gauge block angular alignment is based on monitoring of the interference fringes structure by counting of the number of interference fringes in the lateral and longitudinal section. The method resolution represents the tilt value adequate to the difference between one and two generated interference fringes. In case of two interference fringes in the longitudinal section (gauge block length 30 mm, adequate period of interference fringes *P_longitudinal min_* = 15 mm), the unwanted tilt value is 5 × 10^−6^ rad (0.287 × 10^−3^). In case of two interference fringes in the lateral section (gauge block width 9 mm, adequate period of interference fringes *P_lateral min_* = 4.5 mm), the unwanted tilt value is 17 × 10^−6^ rad (0.974 × 10^−3^). For the gauge block of 100 mm in length, the adequate cosine error 0.001 nm. The algorithm described above is used for gauge block inspection during its length measurement too. In this case, the software monitors the interference fringes structure to detect any unwanted difference in the gauge block position.

## 5. Conclusions

This paper presents methods used for automation of a gauge block calibration process using a technique based on a combination of low-coherence interferometry and laser interferometry. The automation of the measuring process is ensured by several servo loops implementing into a measurement control system. A crucial part of the system is the algorithm for gauge block alignment to the measuring beam which is able to compensate the gauge block lateral tilt up to 0.121 mrad and the longitudinal tilt of the gauge block up to 0.141 mrad. The algorithm is also important for the gauge block position monitoring during its length measurement.
